# A Novel Piecewise Tri-Stable Stochastic Resonance System Driven by Dichotomous Noise

**DOI:** 10.3390/s23021022

**Published:** 2023-01-16

**Authors:** Shuai Zhao, Peiming Shi

**Affiliations:** 1 School of Electrical Engineering, Yanshan University, Qinhuangdao 066004, China; 2 School of Information and Artificial Intelligence, Nanchang Institute of Science & Technology, Nanchang 330108, China

**Keywords:** stochastic resonance, piecewise tri-stable system, dichotomous noise, signal enhancement

## Abstract

Stochastic resonance (SR) has been widely studied as a means of signal processing since its conception. Since SR is different from other denoising methods in nature, it can be used for not only feature extraction but also signal enhancement. Additive white Gaussian noise (AWGN) is often used as a driving source in SR research due to its convenience in numerical simulation and uniform distribution, but as a special noise, it is of great significance to study the SR principle of dichotomous noise as a driving source for nonlinear dynamics. In this paper, the method of piecewise tri-stable SR (PTSR) driven by dichotomous noise is studied, and it is verified that signal enhancement can still be achieved in the PTSR system. At the same time, the influence of the parameters of the PTSR system, periodic signal, and dichotomous noise on the mean of signal-to-noise ratio gain (SNR-GM) is analyzed. Finally, dichotomous noise and AWGN are used as the driving sources of the PTSR system, and the signal enhancement ability and noise resistance ability of the two drivers are compared.

## 1. Introduction

SR theory was proposed in 1981 to explain the glacier problem [1]. For many domains, SR can be used and has good performance [2,3,4,5,6,7,8]. In the aspect of feature extraction and signal enhancement of mechanical signals, SR is used more and more because it is different from other denoising methods [9,10,11,12,13,14,15,16]. Han et al. used colored noise to drive SR, studied the mean first-passage time of the system at this time, and discussed its relationship with the parameters [17]. In Ref. [18], the relationship between the SNR and the depth and width of the potential well is studied, while the relationship to various noises is detailed. Xu et al. used two kinds of noise to drive asymmetric SR, derived some parameter expressions of the system, and studied the SR phenomenon at this time [19]. Sorokin et al. did not use noise but performed parameter excitation on the system and verified that the SR phenomenon could still be generated [20]. In Ref. [21], on the basis of the traditional tri-stable SR, a new dimension is added, a dual-input signal is used, and the bistable SR system is compared with the same situation. Jiao et al. developed a compound multi-stable SR method, also using parametric induction [22]. Shi et al. added the time delay term on the basis of SR, deduced some parameters of the system, and verified its feature extraction capability [23].

As a kind of non-Gaussian noise, it is of great significance to study the SR system driven by dichotomous noise for nonlinear dynamics. Yang et al. studied the under-damped SR system driven by different noises and compared and simulated different situations [24]. In Ref. [25], the cascaded SR is driven by dichotomous noise, and its internal mechanism is studied and applied to feature extraction of bearing signals. Yan et al. used dichotomous noise to drive fractional SR, analyzed its internal mechanism, and derived part of the system formula [26].

In order to make the previously proposed PTSR system more widely applicable, a PTSR method using dichotomous noise as the driving source is proposed. Section 2 introduces the numerical simulation method of dichotomous noise; the PTSR system is driven by it, and signal enhancement is still possible. In Section 3, the influence of the PTSR system parameters, periodic signal parameters, and noise parameters on the SNR-GM is studied. In Section 4, dichotomous noise is compared with the AWGN. The signal enhancement and resistance to noise in the PTSR system are studied when they are the driver. In Section 5, the possibility of further research is discussed. Finally, Section 6 gives the conclusions.

## 2. The PTSR System and Parameters

### 2.1. Numerical Simulation

**Figure 1:**
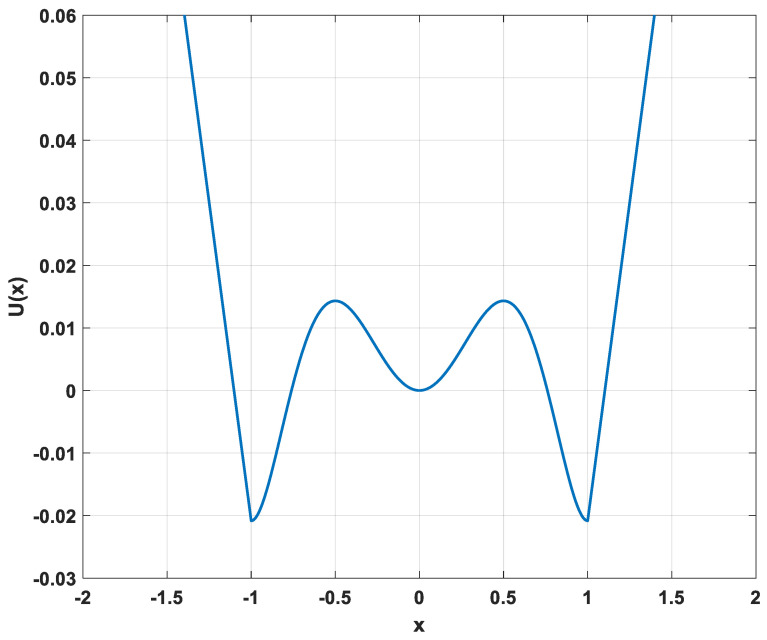
Potential function (m=1,p=0.5,q=1).

In past studies, such as Ref. [27], a PTSR system was proposed, and its potential function is as follows:

(1) $$U\left(x\right)=\{\begin{array}{c} -\frac{3mp^{2}q^{2}\left(p^{2}-q^{2}\right)-m\left(p^{6}-q^{6}\right)}{6\sqrt{2}\sqrt{3q^{2}-p^{2}}-12q}x+\frac{mq^{4}\left(3p^{2}-q^{2}\right)}{12}-\frac{3mp^{2}q^{3}\left(p^{2}-q^{2}\right)-mq\left(p^{6}-q^{6}\right)}{6\sqrt{2}\sqrt{3q^{2}-p^{2}}-12q},x\leq -q\\ \frac{m}{2}p^{2}q^{2}x^{2}-\frac{m}{4}(p^{2}+q^{2})x^{4}+\frac{m}{6}x^{6}\;,\;\;\;\;\;\;-q<x<q\\ \frac{3mp^{2}q^{2}\left(p^{2}-q^{2}\right)-m\left(p^{6}-q^{6}\right)}{6\sqrt{2}\sqrt{3q^{2}-p^{2}}-12q}x+\frac{mq^{4}\left(3p^{2}-q^{2}\right)}{12}-\frac{3mp^{2}q^{3}\left(p^{2}-q^{2}\right)-mq\left(p^{6}-q^{6}\right)}{6\sqrt{2}\sqrt{3q^{2}-p^{2}}-12q}\;,\;x\geq q \end{array}$$

where m>0, p>0 and q>0 are positive number and q>p. Figure 1 displays its potential function.

The SR in the system of Equation (1) in the overdamped state can be expressed as:

(2) $$\frac{dx}{dt}=-\frac{\mathrm{dU}\left(x\right)}{dx}+A\sin\left(2\pi f_{d}t\right)+\xi \left(t\right)$$

where x is the output signal, *A* and $f_{d}$ are the amplitude and the characteristic frequency of periodic signals, ξ(t) is an additive white Gaussian noise (AWGN) satisfying the following conditions:

(3) $$\{\begin{array}{c} \langle \xi \left(t\right)\rangle =0\\ \langle \xi \left(t\right)\xi \left(t-\tau \right)\rangle =2D\delta \left(t\right) \end{array}$$

where τ is the time interval and D is the noise intensity. The PTSR system is said to be driven by dichotomous noise if ξ(t) in Equation (2) is replaced by another type of noise η(t), and η(t) satisfies the following statistical properties:

(4) $$\{\begin{array}{c} \langle \eta \left(t\right)\rangle =0\\ \langle \eta \left(t\right)\eta \left(t^{\prime }\right)\rangle =\frac{D}{\lambda }\exp(-\frac{\left|t-t^{\prime }\right|}{\lambda }) \end{array}$$

where λ is the noise correlation time, and in this case, D can be obtained by the following formula:

(5) $$D=\frac{1}{2}\int_{-\infty }^{+\infty }\left(\langle \eta \left(\tau \right)\eta \left(0\right)\rangle -\langle \eta {(\tau )\rangle }^{2}\right)d\tau =\frac{{\left(a-b\right)}^{2}\mu_{a}\mu_{b}}{{\left(\mu_{a}+\mu_{b}\right)}^{3}}$$

The formula that needs to be explained here is that a and b are the two values set, representing the dichotomous noise between them, while they correspond to the waiting time of $t_{a}$ and $t_{b},$ respectively, then $\mu_{a}=1/t_{a}$, $\mu_{b}=1/t_{b}$. In Refs. [28,29], we can find two probabilities:

(6) $$\{\begin{array}{c} P_{aa}=P(a,t_{n+1}|a,t_{n})=\frac{\mu_{b}}{\mu_{a}+\mu_{b}}+\frac{\mu_{a}}{\mu_{a}+\mu_{b}}\exp(-\left(\mu_{a}+\mu_{b}\right)dt)\\ P_{ba}=P(a,t_{n+1}|b,t_{n})=\frac{\mu_{b}}{\mu_{a}+\mu_{b}}-\frac{\mu_{b}}{\mu_{a}+\mu_{b}}\exp(-\left(\mu_{a}+\mu_{b}\right)dt) \end{array}$$

where $t_{n+1}=t_{n}+dt$, note that dt needs to be set to a small value, which in this article is equal to 0.001.

**Figure 2:**
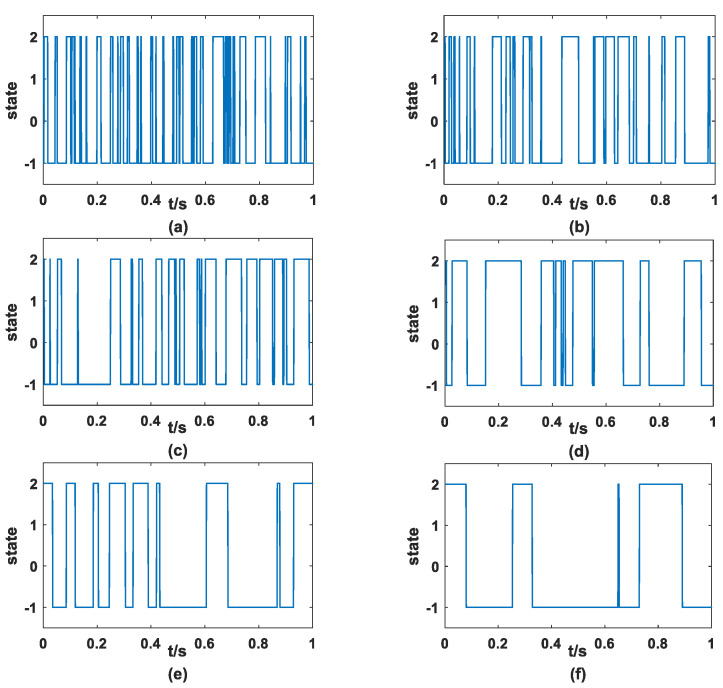
Dichotomous noise (*a* = 2, *b* = −1) (**a**) *D* = 0.01 (**b**) *D* = 0.02 (**c**) *D* = 0.03 (**d**) *D* = 0.04 (**e**) *D* = 0.05 (**f**) *D* = 0.06.

The simulation of dichotomous noise needs to be explained that, if assuming that at the beginning $x_{0}=a$, and then figure out whether the final state $x_{n}$ is equal to a or equal to b, this determines whether $R_{n}$ is relative to $P_{aa}$ or $P_{ba}$. If $R_{n}$ is less than the probability, then $x_{n+1}=a$, otherwise $x_{n+1}=b$. Figure 2 shows the simulated dichotomous noise.

First of all, it can be seen from Figure 2 that dichotomous noise is strictly binary noise, which indicates that the above numerical simulation results are correct. Meanwhile, with the gradual increase in D, the curve representing noise will become sparser, which means that the switching frequency of dichotomous noise between two state values is lower and lower.

### 2.2. Verification by Simulation

After the simulation of dichotomous noise, it is necessary to verify whether the PTSR system can perform feature extraction and signal enhancement under the condition that the PTSR system is driven by dichotomous noise. Since dichotomous noise has little influence on the periodicity of periodic signals, the signal enhancement ability of the PTSR system is mainly verified here. This is to see if the PTSR system can successfully harvest energy from the dichotomous noise and enhance the periodic signal.

**Figure 3:**
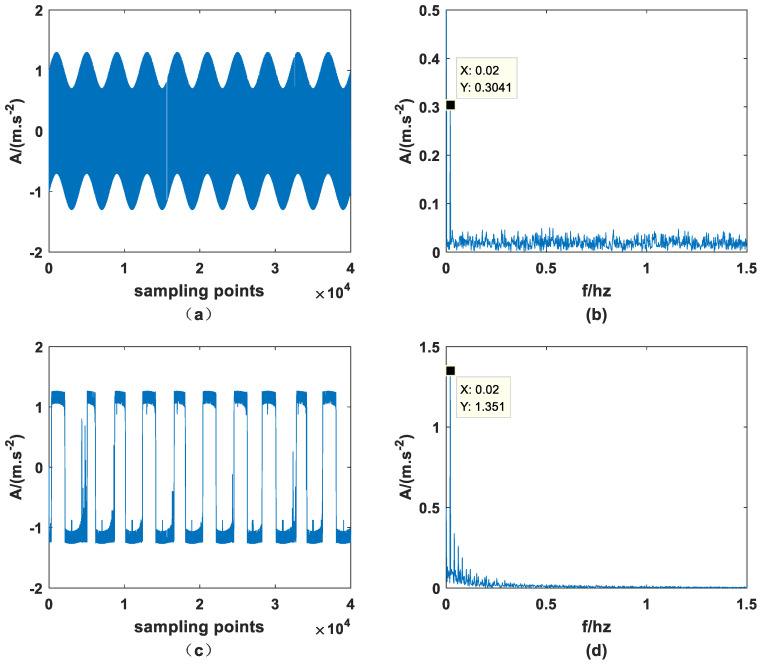
Original signal and output signal: (**a**) input time domain signal, (**b**) input frequency domain signal, (**c**) output time domain signal, (**d**) output frequency domain signal (*m* = 3.0, *p* = 0.5, *q* = 1.2).

Mix a periodic signal of amplitude A=0.3 and characteristic frequency $f_{d}=0.02\;\mathrm{hz}$ with dichotomous noise through the PTSR system and set a=1, b=−1, D=0.5. At the same time, the sampling frequency $f_{s}$ should be set to 80 hz, the number of sampling points should be 40,000, and the step size h should be 0.125. The original signal and the signal processed by the PTSR system are shown in Figure 3.

As previously stated, since dichotomous noise is a binary noise, it is inferior to other noises in the inundation ability of periodic signals. Therefore, although the signal in Figure 3a is not a periodic signal, it can be inferred that it must contain periodic signals. Meanwhile, it can be observed in Figure 3b that the amplitude of characteristic frequency is significantly higher than that of other amplitudes and is approximately equal to 0.3, set before the experiment. It can be found from Figure 3c that the signal is obviously periodic, which indicates that the PTSR system is very suitable for dichotomous noise from the perspective of filter denoising. Finally, the adaptation of the PTSR system to dichotomous noise drivers can be further illustrated by looking at Figure 3d, as the amplitude increases from 0.3041 to 1.351 at the characteristic frequency. This happens because the PTSR system obtains energy from the dichotomous noise and transmits it to the periodic signal.

### 2.3. Dichotomous Noise Drives Different Sr Models

After determining that dichotomous noise can drive PTSR for signal enhancement, comparisons with other SR systems are now being made in order to investigate the suitability of dichotomous noise. In this paper, standard tri-stable SR [30] and classical bistable SR are selected, and their potential functions are $U_{s}\left(x\right)=\frac{m_{s}}{2}p_{s}^{2}q_{s}^{2}x^{2}-\frac{m_{s}}{4}\left(p_{s}^{2}+q_{s}^{2}\right)x^{4}+\frac{m_{s}}{6}x^{6}$ and $U_{c}\left(x\right)=-\frac{c}{2}x^{2}+\frac{d}{4}x^{4}$, respectively. All parameters are set to $m=m_{s}=3,p=p_{s}=0.5,\;q=q_{s}=1.2,\;c=2,\;d=1$, respectively. Next, the characteristic frequency of the periodic signal is unchanged, and the amplitude and noise intensity are increased to 0.5 and 1, respectively. Finally, set a=1, b=−1, respectively. It should be noted that due to the randomness of noise, the average value of 50 repeated experiments was taken. The results are shown in Table 1.

It can be seen from Table 1 that PTSR is the best for dichotomous noise adaptation. Standard tri-stable SR is close to PTSR, but the signal enhancement ability is slightly lower than PTSR. Classical bistable SR can still be enhanced, but the ability is obviously lower than PTSR and standard tri-stable SR.

## 3. SNR-GM of PTSR System Driven by Dichotomous Noise

In this paper, SNR gain ($SNR_{gain}$), proposed in Ref. [31], was used as the standard, and the mean value of the experiment was calculated by repeating 100 times, namely SNR-GM:

(7) $$SNR-GM=\frac{1}{100}\overset{100}{\underset{i=1}{\sum }}SNR_{gain_{i}}$$

where $SNR_{gain}$ is defined as:

(8) $$SNR_{gain}=\frac{SNR_{out}}{SNR_{in}}$$

and $SNR_{in}$ and $SNR_{out}$ are, respectively, shown in the following equation:

(9) $$\{\begin{array}{c} SNR_{in}=\frac{2{\left|Z\left(k_{0}\right)\right|}^{2}}{\sum_{k=0}^{N}{\left|Z\left(k\right)\right|}^{2}-2{\left|Z\left(k_{0}\right)\right|}^{2}}\\ SNR_{out}=\frac{2{\left|Y\left(k_{0}\right)\right|}^{2}}{\sum_{k=0}^{N}{\left|Y\left(k\right)\right|}^{2}-2{\left|Y\left(k_{0}\right)\right|}^{2}} \end{array}$$

$SNR_{in}$ and $SNR_{out}$ are the fast Fourier transforms of the input and output signals, respectively, N is the length of the sequence.

### 3.1. Impact of System Parameters m, p, and q

There are three system parameters in the PTSR system, which are m, p, and q. With the change of these three parameters, the appearance of the potential function PTSR will vary greatly, which is largely due to the movement of particles within the system. Therefore, it is meaningful to study the influence of system parameters on SNR-GM.

**Figure 4:**
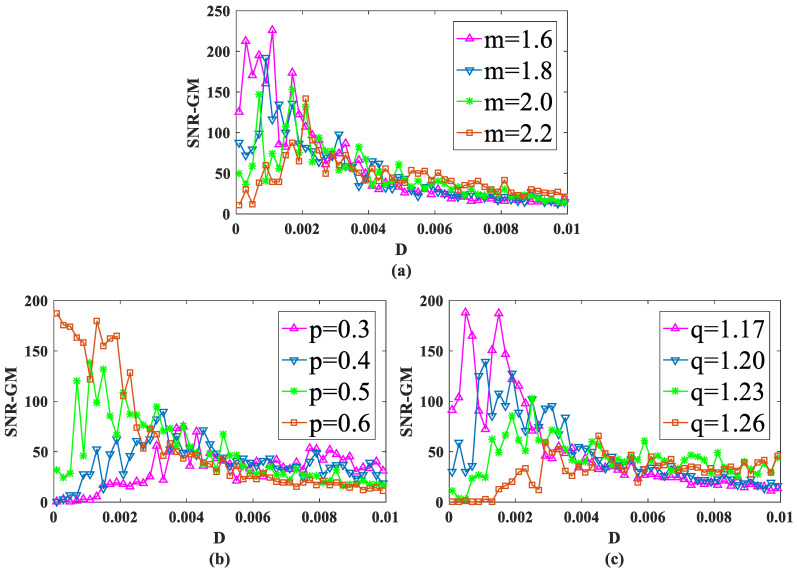
SNR-GM of system: (**a**) Just change *m* (*p* = 0.5, *q* = 1.2), (**b**) Just change *p* (*m* = 2.0, *q* = 1.2), (**c**) Just change *q* (*m* = 2.0, *p* = 0.5).

First, input a periodic signal into the PTSR system and set the amplitude A=0.2 and the characteristic frequency $f_{d}=0.02\;\mathrm{hz}$. Next, set dichotomous noise parameters a and b to 2 and −1, respectively. For experimental parameters, set the overall sampling frequency $f_{d}=80\;\mathrm{hz}$ and step size h to 1/16. After two parameters are fixed and the size of the other parameter is changed, the SNR-GM of PTSR with dichotomous noise as the driving source is drawn in Figure 4.

In Figure 4a–c, all curves rise first and then fall, which is very important as an SR system because the SNR-GM curve in this state can most directly reflect that the SR phenomenon does occur in the system, rather than simply filtering. Then observe the three figures respectively. In Figure 4a, if the value of m is increased, the curve will gradually move to the lower right. This indicates that increasing m will reduce the output SNR-GM, while the noise intensity D, which is most compatible with the PTSR system, will increase. Then, in Figure 4b, the curve presents an opposite trend to that in Figure 4a. With the increase in p, the curve moves upward to the left, indicating that the variation of p increases SNR-GM and decreases the optimal D value at the same time. Finally, it can be concluded from Figure 3c that parameter q and parameter m have roughly the same influence on the system SNR-GM, that is, decrease SNR-GM and increase the adaptive value of D.

**Figure 5:**
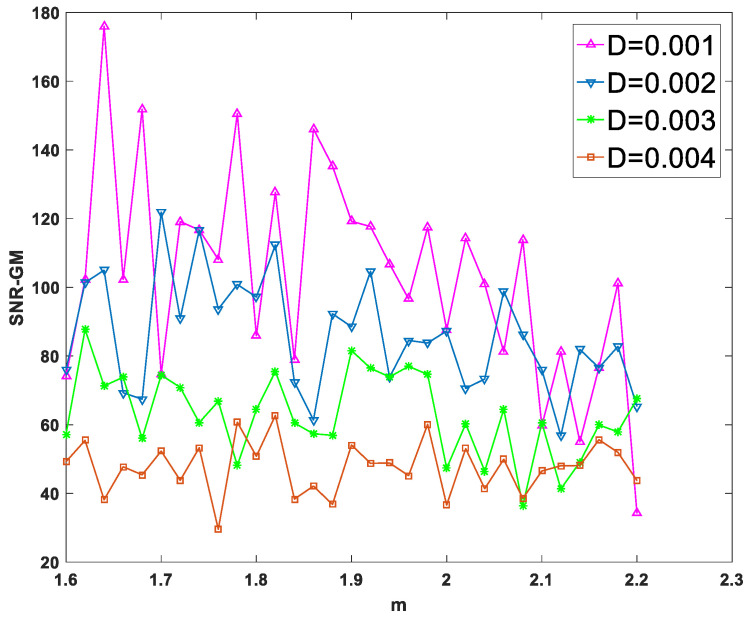
SNR-GM varies with *m* for different values of *D* (*p* = 0.5, *q* = 1.2).

**Figure 6:**
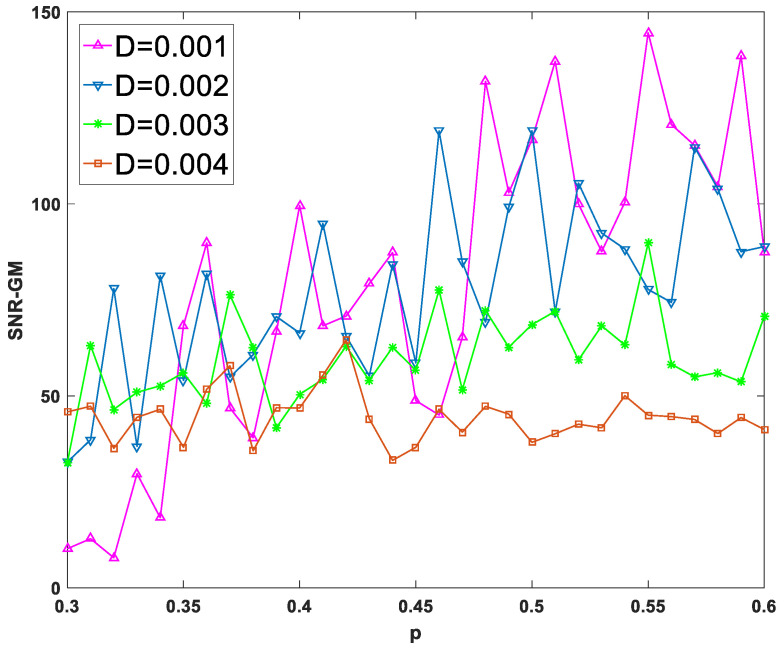
SNR-GM varies with *m* for different values of *D* (*m* = 2.0, *q* = 1.2).

**Figure 7:**
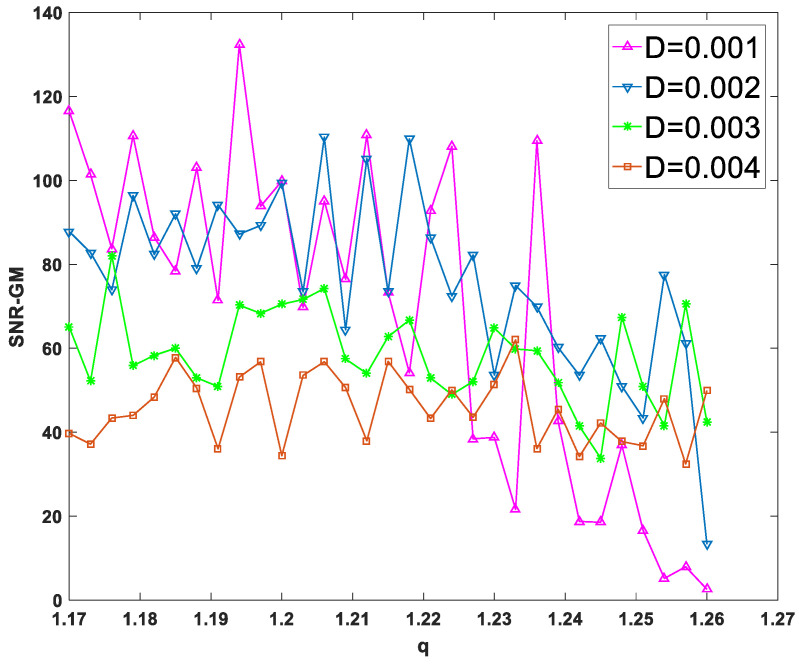
SNR-GM varies with *m* for different values of *D* (*m* = 2.0, *p* = 0.5).

Since the process of noise generation is very random and the effects of PTSR system parameters on the internal system are complex, the above conclusions are further verified. Input the signal with amplitude A equal to 0.3 and characteristic frequency equal $f_{d}=0.02$ hz into the system. Other parameters are set unchanged. This time, the horizontal axis represents the change in parameters, while each experiment changes the size of D, as shown in Figure 5, Figure 6 and Figure 7.

As can be seen from Figure 5 and Figure 7, each curve presents a downward or flat trend, indicating that the increase in m and q has no effect on the increase in SNR-GM in the experiment, which is roughly the same as the previous conclusion that the increase in m or q will lead to the decrease in SNR-GM. Meanwhile, in Figure 6, all curves with different values of D show rising or flat states, which is roughly consistent with the previous conclusion. With the increase in p, SNR-GM will rise or remain unchanged, and SNR-GM will not decline.

### 3.2. Impact of Amplitude A and Characteristic Frequency $f_{d}$ on SNR-GM

**Figure 8:**
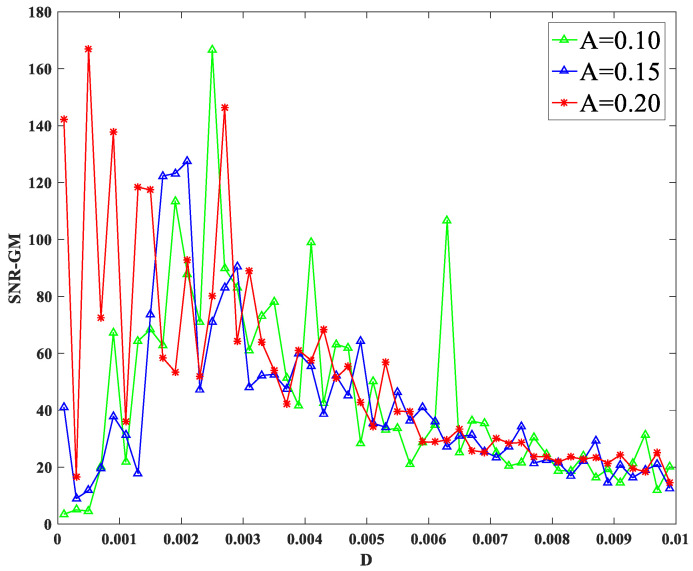
SNR-GM for different values of *A*.

**Figure 9:**
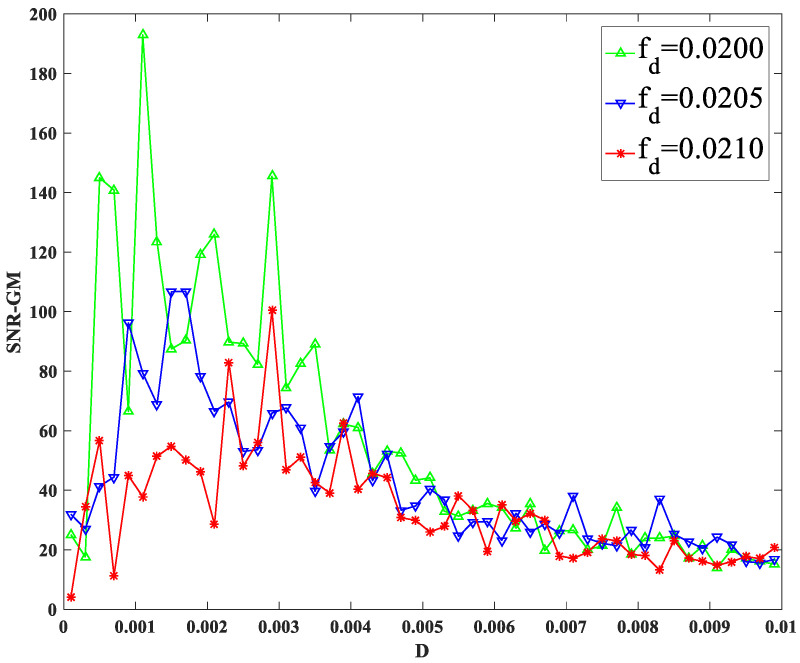
SNR-GM for different values of *f_d_*.

SR is composed of a nonlinear system, random force, and periodic force. Therefore, the influence of periodic signals on SR cannot be ignored. Amplitude and characteristic frequency are two important values in the signal. System parameters m=2.0,p=0.5,q=1.2 were set, while other parameters were consistent with before. The characteristic frequency $f_{d}$ was set as 0.02 hz, and the amplitude A was changed to make the SNR-GM curve of the system, as shown in Figure 8. The amplitude A was set as 0.2, and the characteristic frequency $f_{d}$ of the signal was changed to make the SNR-GM curve of the system, as shown in Figure 9.

As shown in Figure 8, with the change in the amplitude of periodic signal A, the SNR-GM of the system does not change significantly in size, but the position of the SNR-GM curve moves to the left with the increase in A, which indicates that A mainly affects the value of the most suitable noise intensity D in the PTSR system. Then, it is found in Figure 9 that the highest point of the curve moves downward gradually. It can be concluded that with the increase in characteristic frequency $f_{d}$, the SNR-GM of the system will gradually decrease. It should be noted here that because other parameters corresponding to SR generated for different signals vary greatly, $f_{d}$ here can only be adjusted in a very small range each time.

### 3.3. Impact of Dichotomous Noise Parameters a and b on SNR-GM

**Figure 10:**
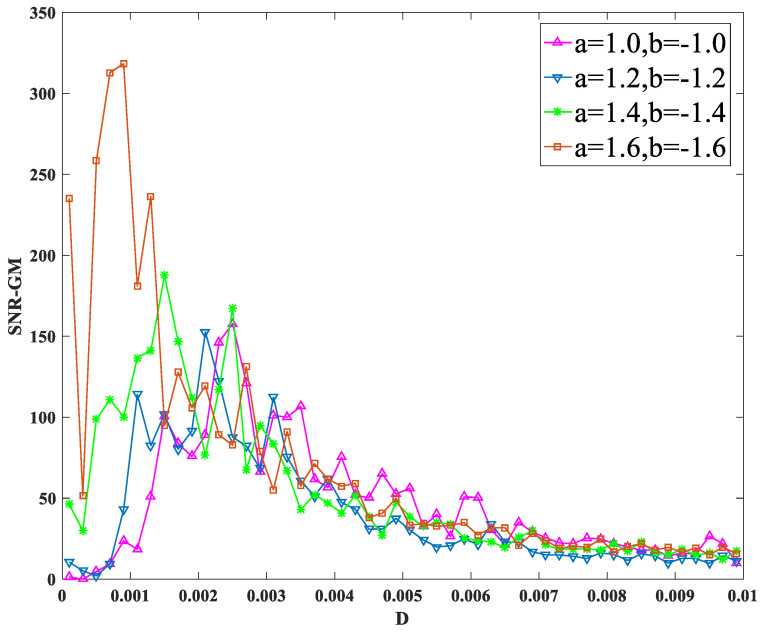
SNR-GM for different values of *a* and *b*.

After studying the parameters of nonlinear system and periodic force, the influence of random force on SNR-GM is now studied. Other parameters remain unchanged. Input signals with amplitude A=0.2 and characteristic frequency $f_{d}=0.02\;\mathrm{hz}$ into the system, and only change the values of a and b each time, as shown in Figure 10.

As can be seen from the figure, SNR-GM gradually moves upward to the left, indicating that increasing the absolute value of a and b will increase the SNR-GM of the system, and the most suitable D in the PTSR system will gradually decrease.

Through the above research, the influence of system parameters, signal parameters, and noise parameters on SNR-GM can be clearly defined. In order to make the conclusion more intuitive, the analysis results are given in Table 2.

## 4. Performance of the PTSR System Driven by Dichotomous Noise Compared with Driven by AWGN

**Figure 11:**
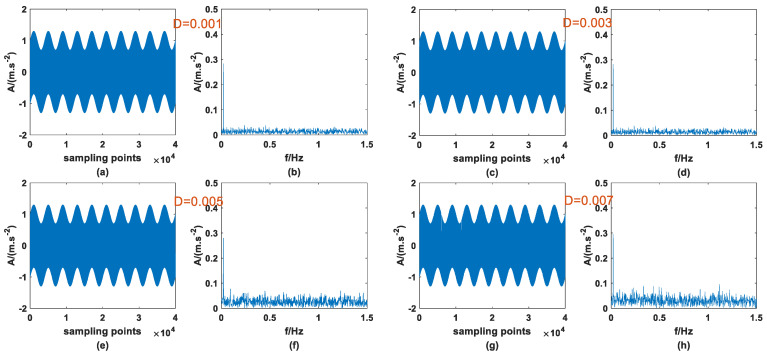
Input signal with dichotomous noise of different *D* (**a**) time domain (*D* = 0.001), (**b**) frequency domain (*D* = 0.001), (**c**) time domain (*D* = 0.003), (**d**) frequency domain (*D* = 0.003), (**e**) time domain (*D* = 0.005), (**f**) frequency domain (*D* = 0.005), (**g**) time domain (*D* = 0.007), (**h**) frequency domain (*D* = 0.007).

**Figure 12:**
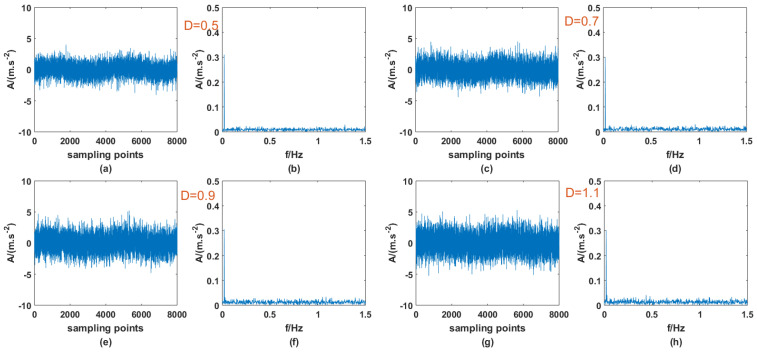
Input signal with AWGN of different *D* (**a**) time domain (*D* = 0.5), (**b**) frequency domain (*D* = 0.5), (**c**) time domain (*D* = 0.7), (**d**) frequency domain (*D* = 0.7), (**e**) time domain (*D* = 0.9), (**f**) frequency domain (*D* = 0.9), (**g**) time domain (*D* = 1.1), (**h**) frequency domain (*D* = 1.1).

**Figure 13:**
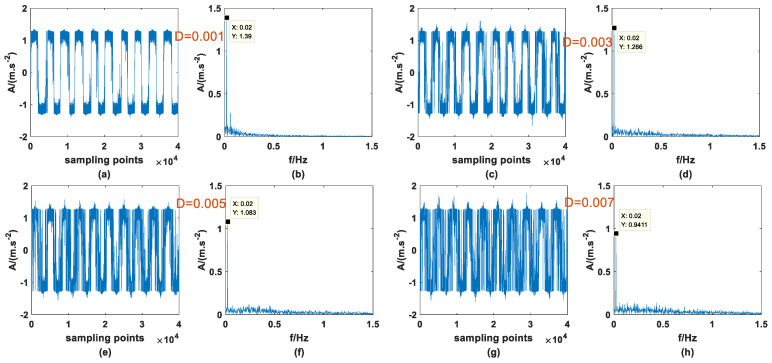
Output signal with dichotomous noise of different *D* (**a**) time domain (*D* = 0.001), (**b**) frequency domain (*D* = 0.001), (**c**) time domain (*D* = 0.003), (**d**) frequency domain (*D* = 0.003), (**e**) time domain (*D* = 0.005), (**f**) frequency domain (*D* = 0.005), (**g**) time domain (*D* = 0.007), (**h**) frequency domain (*D* = 0.007).

**Figure 14:**
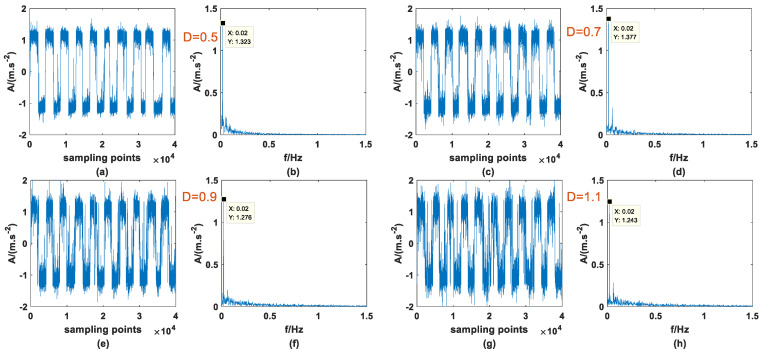
Output signal with AWGN of different *D* (**a**) time domain (*D* = 0.5), (**b**) frequency domain (*D* = 0.5), (**c**) time domain (*D* = 0.7), (**d**) frequency domain (*D* = 0.7), (**e**) time domain (*D* = 0.9), (**f**) frequency domain (*D* = 0.9), (**g**) time domain (*D* = 1.1), (**h**) frequency domain (*D* = 1.1).

Previous studies have shown that SR phenomena can occur in PTSR systems with dichotomous noise as the driving source, and signal enhancement can be successful. At the same time, it was also confirmed in Ref. [27] that AWGN can produce the same effect, so it is meaningful for signal processing to compare the effects of the two types of noise-driving PTSR. The A and $f_{d}$ of the periodic signal are 0.3 and 0.02, the step size *h* is set to 0.8, and the other parameters remain unchanged, then the signal is mixed with the two noises, respectively, and passed through the PTSR system. It should be noted here that because the structure of the two kinds of noise is very different, the interval of the most suitable noise intensity is very different. Here, the appropriate interval is selected for the experiment. Figure 11 and Figure 12 are the input signals, and Figure 13 and Figure 14 are the output signals. By observing Figure 11 and Figure 12, within a certain range, with the increase in D there is no obvious change in the input signal. In addition, characteristic frequency is also the most prominent in the frequency domain, and the amplitude is approximately equal to 0.3. 

Compared with Figure 13 and Figure 14, there is little difference in the signal-strengthening ability between the two noise-driven PTSR systems under suitable D. However, with increasing D, the amplitude of the characteristic frequency in Figure 14 is higher than that in Figure 13, which indicates that the signal-strengthening ability of PTSR systems driven by AWGN is stronger than that driven by dichotomous noise. At the same time, the amplitude of the characteristic frequency in Figure 13 decreases significantly faster than that in Figure 14, indicating that AWGN is more suitable for the utilization of noise energy.

**Figure 15:**
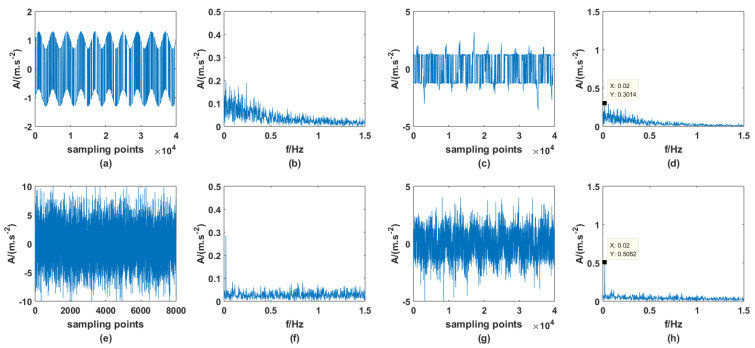
Comparison of high-value D (**a**) input time spectrum with dichotomous noise, (**b**) input frequency spectrum with dichotomous noise, (**c**) output time spectrum with dichotomous noise, (**d**) output frequency spectrum with dichotomous noise, (**e**) input time spectrum with AWGN, (**f**) input frequency spectrum with AWGN, (**g**) output time spectrum with AWGN, (**h**) output frequency spectrum with AWGN.

Next, a relatively high noise intensity D is given for both of the two noises, which are, respectively, 0.05 and 5. The input and output are shown in Figure 15. As can be seen from the figure, when the intensity of noise increases significantly, the dichotomous noise-driven PTSR system has lost the ability of signal strengthening, but the PTSR system driven by AWGN can still perform signal strengthening. This indicates that the energy of AWGN is easier to use and the system is more resistant to AWGN.

## 5. Discussion

In this paper, dichotomous noise is used to drive the PTSR system. It is verified that SR can still occur. The influence of parameters on the PTSR is also discussed and compared with AWGN as the driving source. Next are the specific adaptation rules for the noise intensity dichotomous noise and parameters. Additionally, whether other noises can be used as the driving source of the PTSR system can also be a future research direction. If other noises can also be enhanced using the PTSR, then the ability of these noises to drive the PTSR system should also be compared.

## 6. Conclusions

The following conclusions are obtained through the research:

1. Dichotomous noise as a driving source can still cause SR phenomena in the PTSR system, and dichotomous noise can transfer energy to periodic signals for signal enhancement.
2. Compared with classical bistable SR and standard tri-stable SR, PTSR has higher signal enhancement when dichotomous noise is the driving source.
3. PTSR system parameters m,p,q, periodic signal parameters $A,f_{d}$, and dichotomous noise parameters a,b have an obvious effect on the SNR-GM of the system. The increase in m, q, and $f_{d}$ cause the SNR-GM to show a downward trend, but the effect of p, a, and b is the opposite; amplitude A has little influence on the size of SNR-GM but affects the size of optimal D.
4. PTSR systems have better signal enhancement when they are driven by AWGN, and the range of D adapted is relatively small when driven by dichotomous noise.

## Figures and Tables

**Table 1:** Signal enhancement contrast.

**System**	**Classical Bistable SR**	**Standard Tri-Stable SR**	**PTSR**
The Amplitude of the Enhanced Signal	0.9658	1.41	1.449

**Table 2:** The influence of parameters on SNR-GM.

**Parameters**	m	p	q	A	$f_{d}$	a and b
The change in SNR-GM	reduce	increase	reduce	The increase or decrease is not obvious, which affects the best matching D	reduce	increase

## Data Availability

Not applicable.
